# CD11c^+^ Cells Partially Mediate the Renoprotective Effect Induced by Bone Marrow-Derived Mesenchymal Stem Cells

**DOI:** 10.1371/journal.pone.0072544

**Published:** 2013-08-06

**Authors:** Myung-Gyu Kim, Su Hee Kim, Hyunjin Noh, Yoon Sook Ko, Hee Young Lee, Sang-Kyung Jo, Won Yong Cho, Hyoung Kyu Kim

**Affiliations:** 1 Department of Internal Medicine, Korea University Anam Hospital, Seoul, Korea; 2 Department of Internal Medicine, Gangneung Asan Hospital, Gangneung, Korea; 3 Department of Internal Medicine, Soonchunhyang University Hospital, Seoul, Korea; University of Torino, Italy

## Abstract

Previous studies have shown that induction of immune tolerance by mesenchymal stem cells (MSCs) is partially mediated via monocytes or dendritic cells (DCs). The purpose of this study was to determine the role of CD11c^+^ cells in MSC-induced effects on ischemia/reperfusion injury (IRI). IRI was induced in wildtype (WT) mice and CD11c^+^-depleted mice following pretreatment with or without MSCs. In the in-vitro experiments, the MSC-treated CD11c^+^ cells acquired regulatory phenotype with increased intracellular IL-10 production. Although splenocytes cocultured with MSCs showed reduced T cell proliferation and expansion of CD4^+^FoxP3^+^ regulatory T cells (Tregs), depletion of CD11c^+^ cells was associated with partial loss of MSCs effect on T cells. In in-vivo experiment, MSCs’ renoprotective effect was also associated with induction of more immature CD11c^+^ cells and increased FoxP3 expression in I/R kidneys. However all these effects induced by the MSCs were partially abrogated when CD11c^+^ cells were depleted in the CD11c^+^-DTR transgenic mice. In addition, the observation that adoptive transfer of WT CD11c^+^ cells partially restored the beneficial effect of the MSCs, while transferring IL-10 deficient CD11c^+^ cells did not, strongly suggest the important contribution of IL-10 producing CD11c^+^ cells in attenuating kidney injury by MSCs. Our results suggest that the CD11c^+^ cell-Tregs play critical role in mediating renoprotective effect of MSCs.

## Introduction

Mesenchymal stem cells (MSCs) are multi-potent progenitor cells that can be isolated from various adult tissues and can differentiate into several cell types of mesenchymal origin such as chondrocytes, myocytes, and adipocytes. However, several recent studies have shown the transdifferentiation-independent beneficial effect of MSCs in animal models of ischemic or nephrotoxic acute kidney injury (AKI) [1–4].

MSCs are known to possess an immune-modulatory function through interaction with multiple immune competent cells. Specifically, MSCs can potently inhibit T- and B-cell proliferation from allogeneic or mitogenic stimuli [5–8], and the mechanisms underlying this immune suppressive function are known to be associated with expansion of CD4^+^ Foxp3^+^ regulatory T cells (Tregs) by MSCs [9–12]. In addition, MSCs have also been known to interact with dendritic cells (DCs), making them become regulatory DCs [13–17]. Lymphocytes, DCs and Tregs are all known to participate in the pathogenesis of AKI, raising the possibility that MSCs induced renoprotective effect is partially mediated by their effects on these immune competent cells [18]. Thus, to test a hypothesis that DCs play an important role in MSCs induced attenuation of kidney injury and inflammation, we used CD11c-diphtheria toxin receptor (DTR) transgenic mice. First, we characterized the immunophenotype of in vitro MSC-treated CD11c^+^ cells as well as those from MSC-treated, I/R mice. Subsequently, the renoprotective effect of the MSCs was tested in CD11c^+^ cell-depleted mice, and the effect of adoptive transfer of these cells from wildtype (WT) or IL-10-deficient mice was examined.

## Materials and Methods

### Animals and kidney I/R injury

Male C57BL/6 mice (weight, 20–25 g) aged 6–8 weeks were purchased from Orient (Charles River, Seoul, Korea). The CD11c-DTR B6.FVB-Tg (Itgax-DTR/green fluorescent protein [GFP]; stock number, 004509) and IL-10 knockout (KO; B6.129P2-*Il10* ^tm1Cgn^/J; stock number, 002251) mice were purchased from the Jackson laboratory (Bar Harbor, ME, USA). All experimental protocols were approved by the animal care committee of Korea University and followed the NIH publication ‘Principles of Laboratory Animal Care’. The mice were anesthetized and subjected to bilateral renal pedicle clamping for 32 min. After the clamps were removed, the mice were observed for 24 hours (h) and sacrificed for blood and tissue analysis.

### MSC culture and administration

The MSCs were prepared from bone marrow as previously described [19]. The mouse bone marrow cells were seeded in flasks at a concentration of 1 × 10^6^/75 cm^2^ in M10 medium (DMEM medium containing 10% fetal calf serum (FCS), 1% penicillin-streptomycin, and 1% l-glutamine), and all non-adherent cells were removed after 24 h. The medium was replenished every 3 days and after at least 5 passages, The MSCs were isolated and analyzed for surface expression of stem cell antigen-1 (Sca-1), CD34, and CD45. For further characterization of the MSCs, we assessed the ability of these cells to differentiate into adipocytes and osteocytes (data not shown). In addition, MSC-conditioned medium was collected after the second passage of MSCs, cultured for 48 h at a concentration of 1 × 10^6^/75 cm^2^, and filtered through a 0.22-µm–filtration unit (Millipore, Bedford, MA, USA). The mice were administered 1 × 10^6^ MSCs or MSC-conditioned medium (200 µL) intraperitoneally at 4 h before renal I/R; the control mice were injected with phosphate-buffered saline (PBS; 200 µL). In some experiments using MSCs, mice were injected with 200 µg of neutralizing anti–mouse IL-10 Abs (BioXCell) or isotype IgG intraperitoneally at 4 h before renal I/R with MSCs.

### Biochemical and histological analyses

Serum creatinine levels were measured using a Hitachi 747 automatic analyzer. Tubular injury was semi-quantitatively assessed in periodic acid-Schiff (PAS)-stained kidney sections, as previously described [18]. The number of terminal deoxynucleotidyl transferase dUTP nick end labeling (TUNEL)-positive apoptotic cells was counted. We stained the formalin-fixed, paraffin-embedded kidney sections with monoclonal antibodies against F4/80 (Serotec, Kidlington, UK), CD11c (Abcam, Cambridge, MA, USA), and Ly6G (BD Biosciences, San Diego, CA, USA). A total of 8–10 high power fields (HPFs) were captured, and the mean number of positive cells was compared.

### Quantification of cytokines and chemokines by cytometric bead array

The cytokines and chemokines in the kidney tissues and culture supernatants were quantified using a cytometric bead array (CBA). A mouse inflammation kit (BD Biosciences) was used according to the manufacturer’s instructions to simultaneously detect mouse interleukin-12p70 (IL-12p70), tumor necrosis factor-α (TNF-α), interferon-γ (IFN-γ), monocyte chemoattractant protein-1 (MCP-1), IL-10, and IL-6, as previously described [18].

### Fluorescence-activated cell sorting analysis of spleen and kidney CD11c^+^ cells

Spleen-derived CD11c^+^ cells were isolated by magnetic-assisted cell sorting (MACS) (Milteny Biotech, Bergisch Gladbach, Germany). The purity of the isolated CD11c^+^ cells was 90%. To determine the effect of the MSCs on the CD11c^+^ cell phenotype, we performed a trans-well experiment. Briefly, the CD11c^+^ cells (0.5 × 10^6^ cells) were directly seeded onto MSCs (3.3 × 10^5^) in the lower trans-well system, or separated from the MSCs in the upper trans-well system, in RPMI 1640 medium supplemented with 5% FCS at 37° C in 5% CO2. After 3 days of culture, the CD11c^+^ cells were harvested and the surface expressions of CD11b; CD11c; maturation marker, CD80 were examined by flow cytometry. For the analysis of intracellular TNF-α and IL-10, stained cells were additionally incubated with 1% FACS permeabilizing solution, followed by phycoerythrin (PE)-conjugated anti- IL-10 or TNF-α (eBioscience, San Diego, CA, USA). All the in vitro experiments were performed in triplicate and repeated 3 times. Additionally, the immunophenotype and TNF-α and IL-10 production levels were examined from the CD11c^+^ cells isolated from the MSC- or vehicle-treated I/R mice. For flow cytometric analysis of kidney leukocytes, kidney tissues were processed as previously described [18]. The antibodies (CD45, CD11c, CD11b, and CD80) were purchased from eBioscience (San Diego, CA, USA) and were incubated with fresh kidney suspensions. Four-color fluorescence flow cytometric analyses were performed (FACSCalibur^TM^; BD Bioscience), and the data were analyzed using FlowJo program (Tree Star Inc., Ashland, CA, USA).

### Real time RT-PCR

For detection of FoxP3 mRNA expression in kidney, real time RT PCR was performed using an iCycler IQ Real time PCR Detection System (Bio-Rad, Hercules, CA, USA), the SYBR Supermix kit (Bio-Rad) and the RT2 PCR Primer Set for FoxP3 (Invitrogen, Life Technologies). The reference gene (RT2 PCR Primer Set, Applied Biosystems) was 18s.

### In vivo depletion of CD11c^+^ cells

To deplete the CD11c^+^ cells, diphtheria toxin (DT; 4 ng/g) was intraperitoneally administered to the CD11c-DTR transgenic mice 18 h before I/R. The MSCs were intraperitoneally administered 4 h before I/R, and the plasma creatinine levels and kidney histology obtained 24 h after I/R were compared.

### Adoptive transfer experiments

The spleen suspensions were filtered through 100-µm BD Falcon cell strainers and purified by density centrifugation using Ficoll at 90 ×*g* for 20 min. The cell suspension from the low-density interface was incubated with a cocktail of biotin-conjugated monoclonal antibodies against CD90, CD45R, CD49b, CD8a, CD3, and Ly-6G (Miltenyi Biotec) and negatively selected. The isolated splenic cells were positively selected for the CD11c^+^ cell population. The purity (>90%) of the CD11c^+^ cells was determined using flow cytometry. The CD11c^+^ cells (1 × 10^6^) from the WT mice and IL-10 KO mice were adoptively transferred into the CD11c^+^-DTR transgenic mice after the DT (4 ng/g) injection and 1 × 10^6^ MSCs were intraperitoneally administered 4 h before kidney I/R.

### MSC-splenocyte co-culture and cell proliferation assay

For the co-culture experiments, the MSCs were first plated in 96-well plates (BD Biosciences) at a density of 1 × 10^4^ cells per well in 100 µL of complete medium consisting of RPMI-1640 supplemented with 10% FBS, 100-U/mL penicillin, and 10-mg/mL streptomycin. To prevent nonspecific proliferation of the MSCs by anti-CD3 and anti-CD28 stimulation, the MSCs were pretreated with mitomycin C (50 µg/mL) and washed 5 times before plating; no significant proliferation of the MSCs was detected. Following this, 1 × 10^5^ splenocytes from the C57/BL6 mice and CD11c^+^ cell-depleted mice were added, and their proliferative responses against the mouse anti-CD3 and anti-CD28 antibodies (BD BioCoat^TM^ Mouse T-cell Activation Plates; BD Biosciences) in the presence or absence of MSCs were determined. The proliferation was quantified by labeling the cells with 5-bromodeoxyuridine (BrdU) for 18 h, and then, determining the amount of BrdU incorporated after 72 h of culture. In another experiment to assess the effect of CD11c^+^ cells in mediating MSCs-induced Treg expansion, total splenocytes from WT or CD11c-DTR mice were cocultured with MSCs at a 10:1 ratio (splenocyte : MSC) with low-dose recombinant IL-2 (10U/ml) and after 72 h of coculture, CD4^+^Foxp3^+^ Treg was analyzed by flow cytometry.

### Labeling of MSCs and CD11c^+^ DCs with DiI and in vivo tracking

For in vivo trafficking of administered MSCs or CD11c^+^ DCs, the cells were isolated and labeled with CellTracker^TM^ CM-DiI (Invitrogen, Carlsbad, CA, USA) at a final concentration of 25-mg/mL DiI at 37° C for 15 min and injected as previously described. At 24 h following I/R, the mice were perfusion-fixed with 4% paraformaldehyde, and the kidney, lung, spleen, liver, and peritoneum were sequentially fixed and frozen at 70° C. Fluorescence microscopy was performed to analyze the 4–8-µm cryosections using a rhodamine filter to identify the MSCs and CD11c^+^ DCs.

### Statistical Analysis

All data are presented as the mean ± standard error (SE) and were analyzed by the Kruskal–Wallis test. A p value less than 0.05 was considered statistically significant.

## Results

### MSCs and MSC-conditioned culture media attenuated kidney injury and suppressed inflammation in I/R-induced AKI

**Figure 1:**
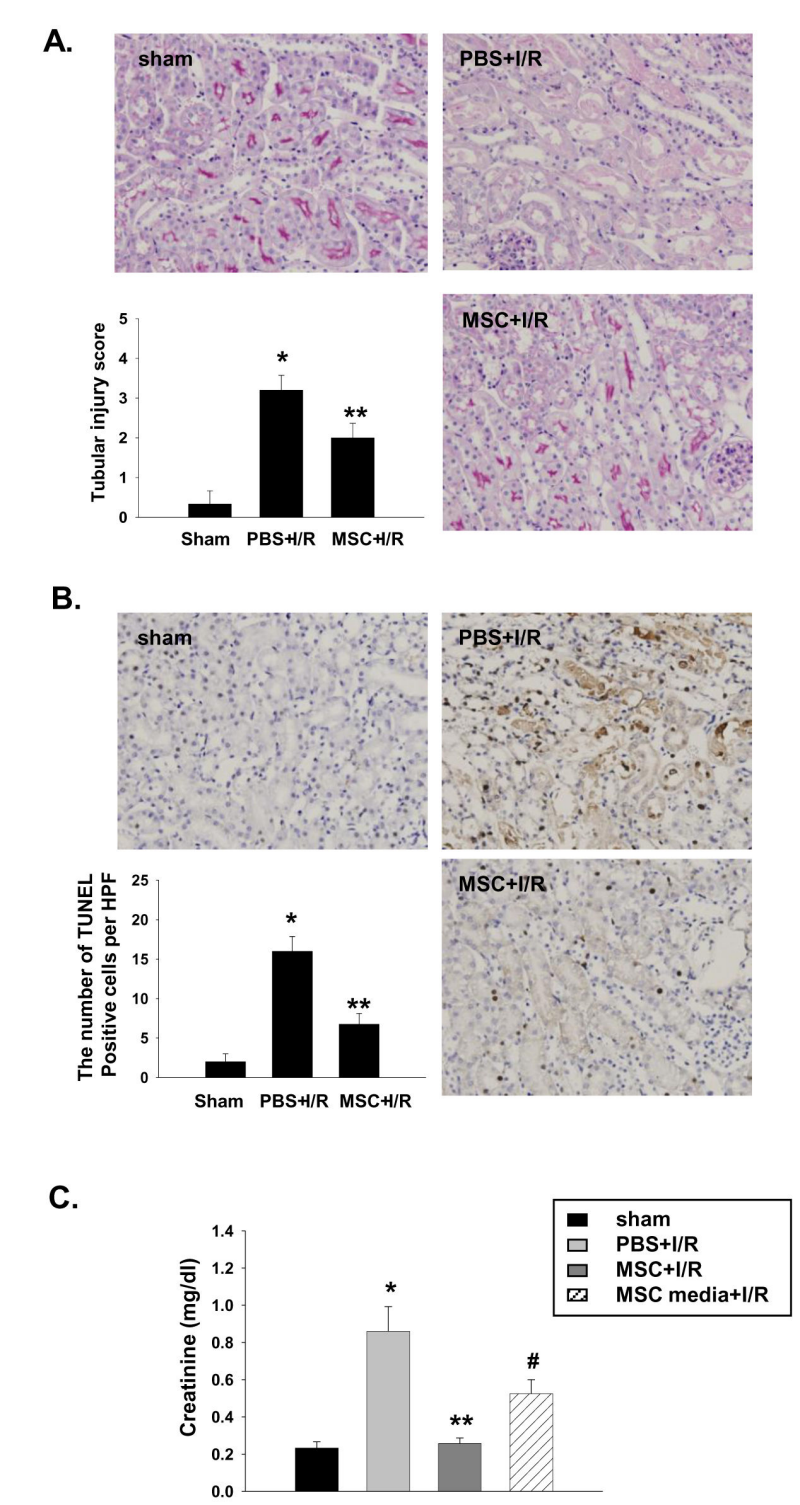
MSCs and MSC-conditioned culture media (MSC-CM) pretreatment attenuated kidney injury in I/R-induced AKI. The MSCs (1 × 10^6^) or MSC-CM was intraperitoneally administered 4 h before I/R. The kidney function and histologic injury were assessed. (A) Kidney histology, PAS staining, magnification ×200. Tubular injury was semi-quantitatively scored as previously reported [18]. (B) TUNEL-positive cells, magnification ×200. The mean numbers of apoptotic cells per HPF (total 8–10 HPFs) were compared. (C) Serum creatinine, (n = 5–6 per group), * p < 0.05 compared to the sham, ** p < 0.05 compared to PBS + I/R, # p < 0.05 compared to MSC + I/R.

**Figure 2:**
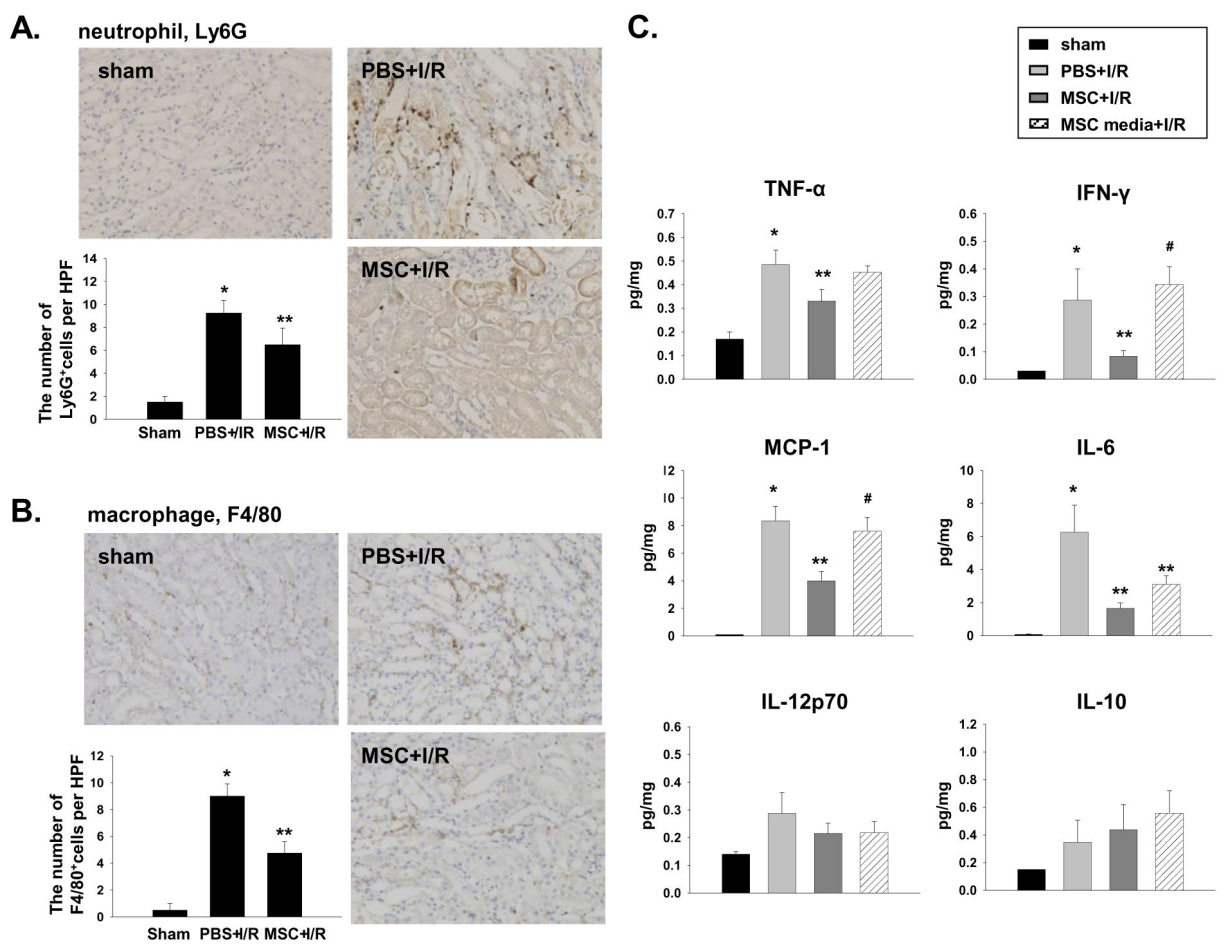
MSC pretreatment attenuated kidney inflammation in I/R-induced AKI. The MSCs (1 × 10^6^) were intraperitoneally administered 4 h before I/R and the kidney inflammation was assessed by immunohistochemical detection of neutrophils and macrophages, and kidney cytokine concentration. (A) Neutrophil (Ly6G) immunohistochemistry, magnification ×200. (B) Macrophage (F4/80) immunohistochemistry, magnification ×200. (C) The kidney cytokine concentration was measured by CBA (n = 5–6 per group). * p < 0.05 compared to the sham, ** p < 0.05 compared to PBS + I/R, # p < 0.05 compared to MSC + I/R.

**Figure 3:**
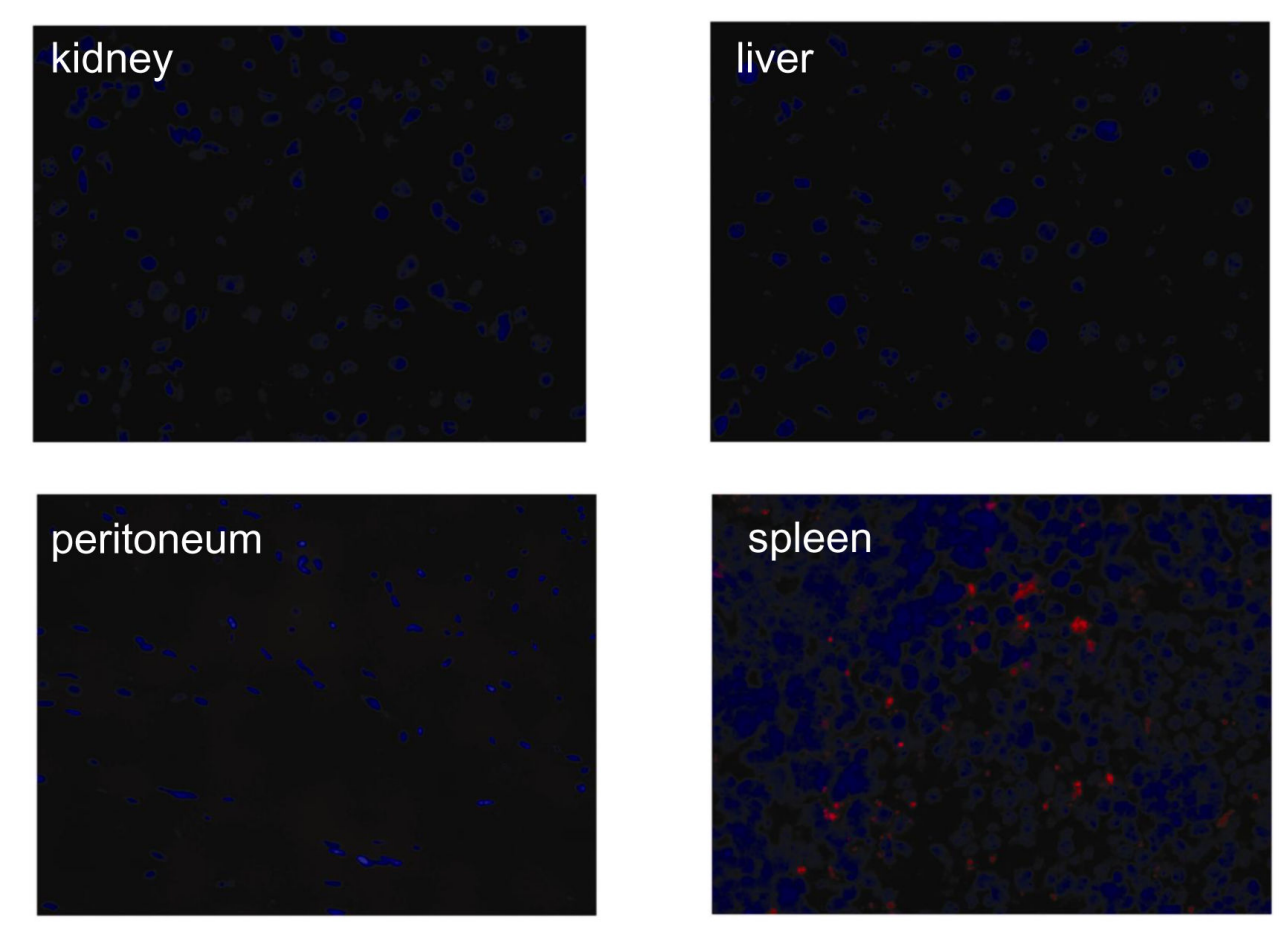
In-vivo trafficking of DiI-labeled MSCs in I/R-induced AKI. The cultured MSCs were labeled with CellTracker ^TM^CM-DiI and intraperitoneally injected into mice 4 h before I/R. At 24 h after I/R, the mice were perfusion-fixed with 4% paraformaldehyde and the kidneys, lungs, spleens, livers, and peritoneum were fixed with 5% sucrose in PBS overnight at 4° C, 30% sucrose in PBS for 8 h at 4° C, and frozen at -70° C. Fluorescence microscopy was performed on the 4–8-µm cryosections using a rhodamine filter to identify the MSCs, magnification ×200.

As expected, pretreatment with MSCs markedly attenuated functional and histologic kidney injury at 24 h after I/R. The kidneys from the MSC-treated mice showed significantly less tubular injury than that from the vehicle-treated mice, which had extensive tubular injury characterized by tubular cell necrosis, dilation of tubules, and cast formation in the outer medulla (Figure 1A, C). The number of TUNEL-positive apoptotic tubular cells was also decreased in MSC pretreated mice (Figure 1B). In addition, the MSC pretreatment induced a strong anti-inflammatory effect assessed by Ly6G and F4/80 immunohistochemical staining (Figure 2A, B). The kidneys from vehicle-treated mice showed increased levels of TNF-α, IFN-γ, MCP-1, and IL-6, whereas the measured cytokine levels in the MSC-treated mice were significantly decreased (Figure 2C). In the trafficking experiment, the administered MSCs were occasionally found in the spleen, but not in the kidney, liver, or peritoneum (Figure 3). In addition, the renoprotective effect was observed in mice treated with the MSC culture media alone. However, the magnitude of protection was significantly lesser than that observed in the direct MSC treatment (Figures 1C and 2C).

### MSCs induce aberrant CD11c^+^ population

**Figure 4:**
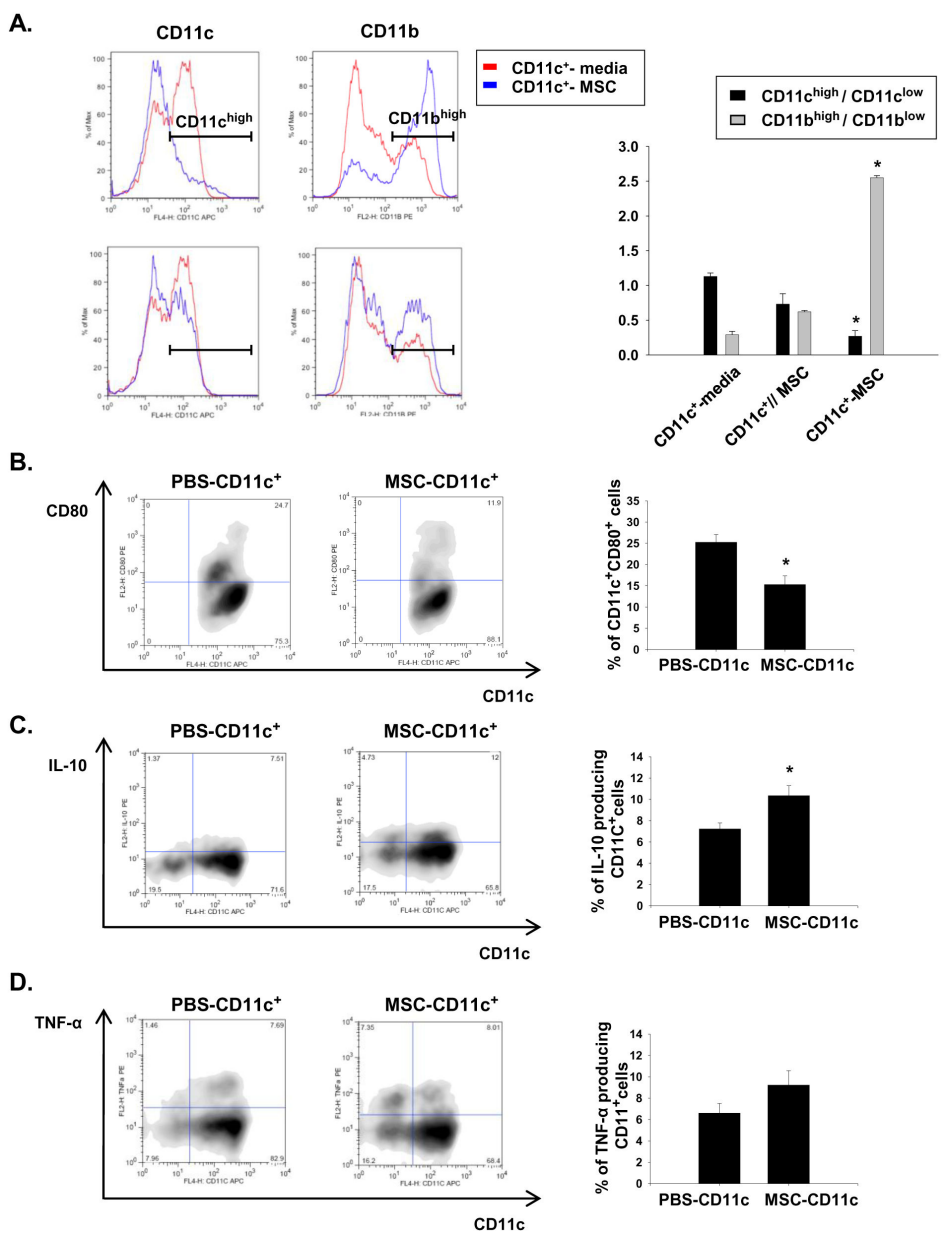
In vitro co-culture with MSCs induced a phenotypic change in the CD11c^+^ cells. The spleen-derived CD11c^+^ cells were isolated by magnetic sorting and co-cultured with the vehicle (media) or MSCs either in direct contact or separated by trans-well insets for 72 h. Subsequently, their phenotypes were assessed by flow cytometry. (A) Left, expression of CD11c; right, expression of CD11b. CD11c-MSC, same chamber; CD11c//MSC, lower/upper separation. The isolated CD11c^+^ cells can be divided into CD11c^high^ or CD11c^low^ and CD11b^high^ CD11b^low^ populations. The phenotypic changes were semi-quantitatively assessed by calculating and comparing the ratios of cell types (CD11c^high^/CD11c^low^ or CD11b^high^/CD11b^low^). * p < 0.05 compared to CD11c^+^//media. (B) Expression of the maturation marker CD80 in the CD11c^+^ cells. (C) Intracellular IL-10 in the CD11c^+^ cells. (D) Intracellular TNF-α in the CD11c^+^ cells. * p < 0.05 compared to the PBS-treated CD11c^+^ cells. All in vitro experiments were performed in triplicate and repeated 3 times.

To assess the effect of MSCs on phenotype of CD11c^+^ cells, we performed in vitro coculture experiment. Splenic CD11c^+^ cells were isolated using MACS beads. We found that the CD11c^+^ cells directly co-cultured with MSCs acquired an altered phenotype that was characterized by reduced CD11c expression and increased myeloid lineage marker CD11b expression. This phenotypic change was far less significant when the cells were physically separated from the MSCs (CD11c//MSCs) (Figure 4A). The CD11c^high^/CD11c^low^ ratio was significantly decreased following co-culture with MSCs, whereas the CD11b^high^/CD11b^low^ ratio was increased. Furthermore, the percentage of CD11C^+^ cells that co-expressed the maturation marker CD80 decreased when they were co-cultured with MSCs (Figure 4B). In addition, the intracellular cytokine staining showed a significantly higher percentage of IL-10-producing CD11c^+^ cells in the MSCs-treated cells than that in the PBS-treated cells, while no difference was observed in the percentages of CD11c^+^ cells expressing TNF-α (Figure 4C, D). All these results could suggest that the MSCs induced aberrant CD11c^+^ cells that possess a more immature, regulatory phenotype.

### Phenotypic changes in CD11c^+^ cells in the MSC-treated I/R mice

**Figure 5:**
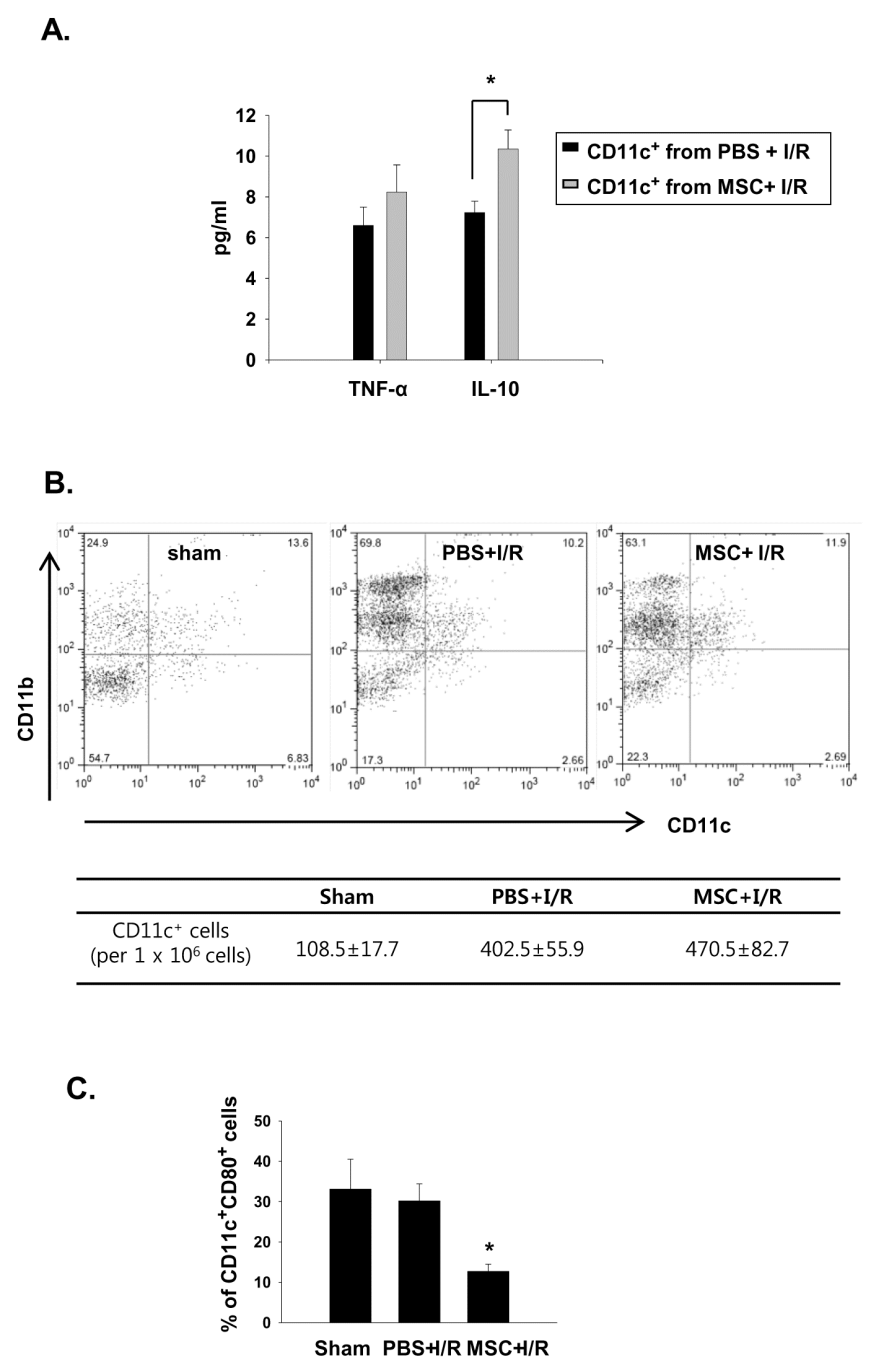
CD11c^+^ cells in the MSC-treated I/R mice showed phenotypic changes. The mice were pretreated with 10^6^ MSCs or vehicle (PBS) for 4 h before I/R. The mice were then sacrificed and analyzed. (A) Cytokine production from the spleen CD11c^+^ cells. (B) Flow cytometric analysis and number of kidney CD11c^+^ cells in I/R or MSC + I/R kidneys. (C) The percentage of mature DCs (expressing CD80) in kidney CD11c^+^ cells. n = 5–6 per group, * p < 0.05 compared to PBS + I/R.

We also analyzed the phenotype of the CD11c^+^ cells from the vehicle- or MSC-treated I/R mice. The CD11c^+^ cells isolated from the spleens of the MSC-treated I/R mice produced significantly greater amounts of IL-10 than those of the vehicle-treated I/R mice, whereas no difference was observed in the TNF-α levels (Figure 5A). In the analysis of kidney DC phenotype, there was no significant difference in the total number of CD11c^+^ cells in kidneys between the groups (Figure 5B). However, the percentage of mature DCs (expressing CD80) decreased significantly in kidneys of MSC-treated I/R mice compared to that of vehicle-treated mice (Figure 5C).

### Depletion of the CD11c^+^ cells abrogated the MSC-induced suppression of lymphocyte proliferation, expansion of Tregs

**Figure 6:**
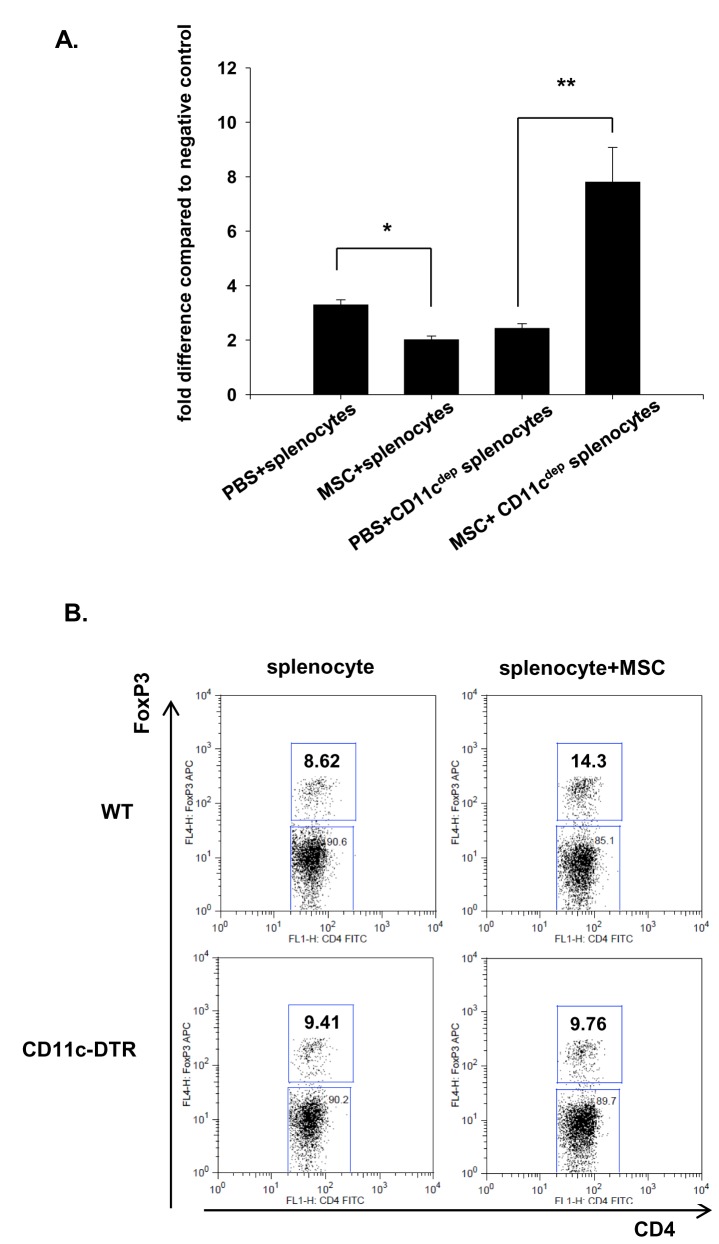
Depletion of the CD11c^+^ cells abrogated the effect of the MSCs on T-cell proliferation and expansion of CD4^+^FoxP3^+^ cells. (A) The splenocytes (1 × 10^5^) from the WT mice and CD11c^+^ cell-depleted mice were stimulated by anti-mouse CD3 and CD28 antibodies in the presence of PBS or MSCs (1 × 10^4^) for 72 h. The MSCs were pretreated with mitomycin-C (50 µg/mL) to prevent their own proliferation. The cells were labeled with BrdU for the final 18 h and the amount of BrdU incorporated was determined. The fold difference compared with the negative control is expressed (n = 4–5 per group). * p < 0.05 compared to PBS + splenocytes, ** p < 0.05 compared to PBS + CD11c^dep^ splenocytes. (B) Total splenocytes from WT or CD11c-DTR mice were cocultured with MSCs at a 10:1 ratio (splenocyte : MSC) with low-dose recombinant IL-2 (10U/ml) for 72 h and the CD4^+^FoxP3^+^ cell population was analyzed by flow cytometry.

Splenocytes cultured in the presence of anti-CD-3 and anti-CD28 showed proliferation of T-lymphocytes. However, co-culturing the splenocytes with the MSCs significantly inhibited the T-lymphocyte proliferation, and this anti-proliferative effect was completely abrogated when the CD11c^+^ cell-depleted splenocytes from the CD11c-DTR transgenic mice were used (Figure 6A). We also observed that co-culture of the splenocytes with the MSCs resulted in expansion of the CD4^+^ FoxP3^+^ Tregs. However, the expansion of Tregs was also partially suppressed by depletion of CD11c^+^ cells (Figure 6B).

### The MSCs renoprotective effect was partially abrogated in the CD11c^+^-depleted mice

**Figure 7:**
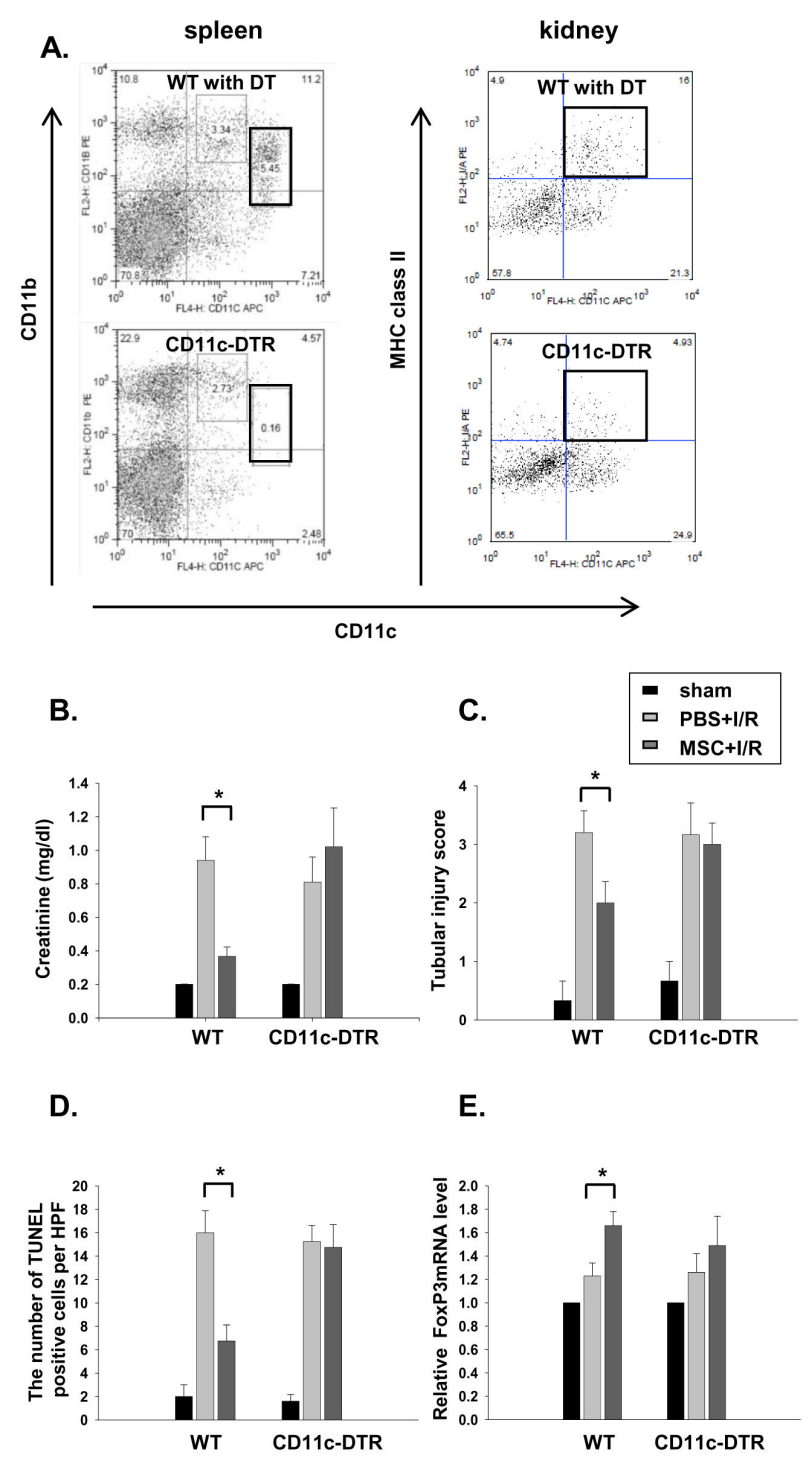
The beneficial effect of the MSCs was completely abrogated in CD11c^+^ cell-depleted mice. For CD11c^+^ cells depletion, DT (4 ng/g) was intraperitoneally administered to CD11c-DTR transgenic mice and WT mice. At 18 h later, flow cytometric analysis of the spleen and kidney CD11c^+^ cells was performed. (A) Depletion of spleen CD11c^high^ CD11b^+^ DCs and kidney CD11c^+^ MHC II^+^ cells was confirmed. (B) Serum creatinine, (C) the tubular injury score, (D) the mean numbers of apoptotic cells per HPF and (E) FoxP3 mRNA expression in kidney were measured at 24 h after renal I/R in MSC- or PBS-pretreated mice (WT and CD11c-DTR transgenic mice). n = 6–7 per group, * p < 0.05 compared to PBS + I/R.

Pretreatment of the transgenic mice with the human diphtheria toxin (4 ng/g) resulted in a significant depletion of GFP^+^ cells, especially the CD11c^high^ CD11b^+^ cells in the spleen and CD11c^+^ MHC II^+^ cells in the kidney (Figure 7A and Figure S1). Although depletion of CD11c^+^ cells in transgenic mice did not have an impact on kidney dysfunction following IRI, the renoprotective effect of the MSCs in I/R injury was partially abrogated in CD11c^+^ cell-depleted mice (CD11c^dep^ + MSC + I/R; Figure 7B–D). In addition, we also found that increased FoxP3 mRNA expression in kidneys of MSC treated IR mice was partially suppressed when CD11c^+^cells were predepleted using CD11c DTR transgenic mice, suggesting the important contribution of CD11c^+^ cells as well as Tregs in the renoprotection by MSC (Figure 7E).

### Adoptive transfer of IL-10-deficient CD11c^+^ cells failed to restore the renoprotective effect of the MSCs

**Figure 8:**
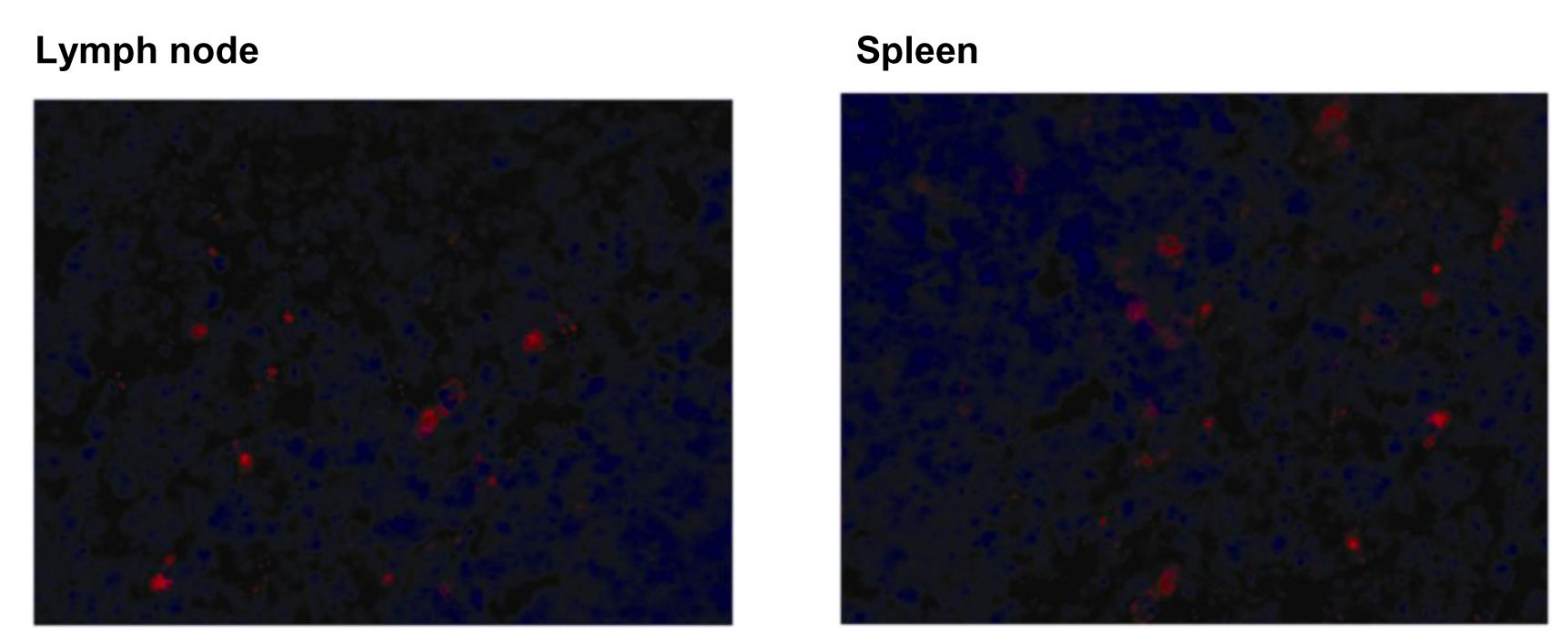
Adoptively transferred DiI-labeled CD11c^+^ cells in I/R-induced AKI. The spleen-derived CD11c^+^ cells were isolated and labeled with CellTracker ^TM^CM-DiI. The cells were then intraperitoneally injected into mice 4 h before I/R. At 24 h after I/R, the mice were perfusion-fixed with 4% paraformaldehyde and the kidney, lung, spleen, liver, and lymph nodes were sequentially fixed with 5% sucrose in PBS overnight at 4° C, 30% sucrose in PBS for 8 h at 4° C, and frozen at -70° C. Fluorescence microscopy was performed on the 4–8-µm cryosections using a rhodamine filter to identify infused CD11c^+^ cells, magnification ×200.

To gain a better insight into the role of CD11c^+^ cells, we adoptively transferred the CD11c^+^ cells following depletion to the CD11c-DTR transgenic mice. The adoptively transferred CD11c^+^ cells were occasionally found in the lymph nodes and spleen, but not in the kidney (Figure 8). Transferring CD11c^+^ cells partially restored the renoprotective effect of the MSCs in CD11c^+^ depleted I/R mice.

**Figure 9:**
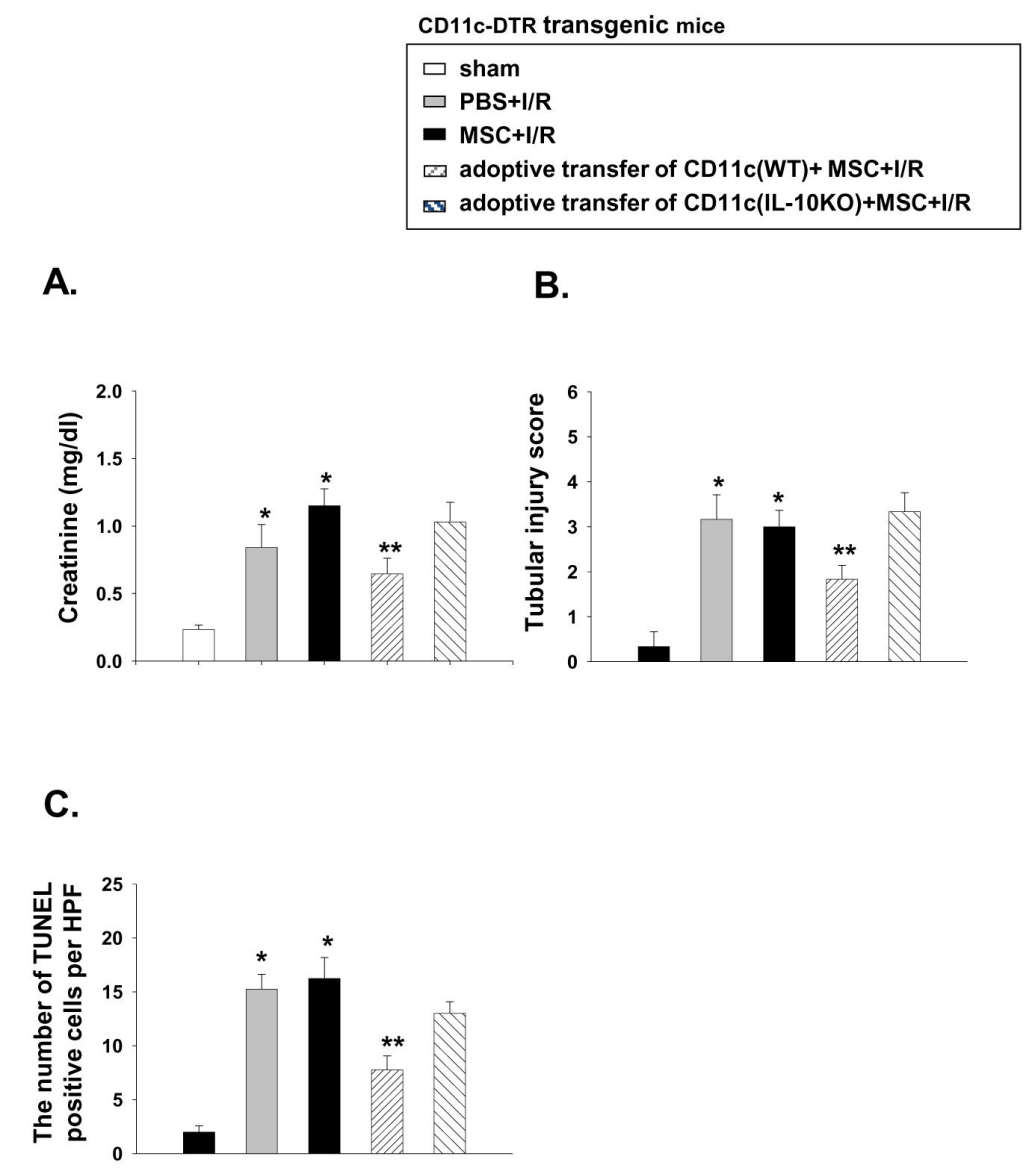
Adoptive transfer of IL-10-deficient CD11c^+^ cells failed to restore the renoprotective effect of the MSCs. The CD11c^+^ cells were isolated from the WT and IL-10 KO mice and adoptively transferred into the CD11c^+^-DTR transgenic mice after DT (4 ng/g) injection (1 × 10^6^ cells). One million MSCs were then intraperitoneally administered 4 h before renal I/R and the mice were then sacrificed and (A) the serum creatinine levels, (B) the tubular injury and (C) the mean numbers of apoptotic cells per HPF were measured at 24 h after renal I/R injury (n = 4–5 per group). * p < 0.05 compared to sham, ** p < 0.05 compared to MSC + I/R in the CD11c-DTR transgenic mice.

**Figure 10:**
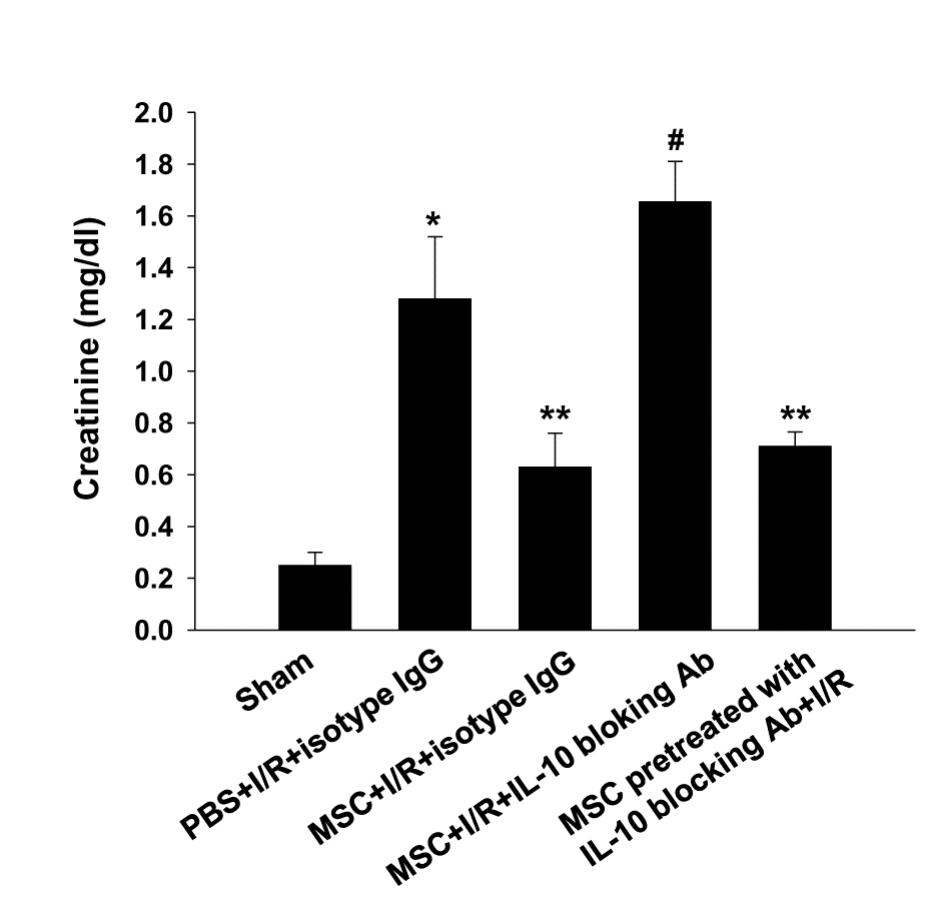
IL-10 blocking Ab abrogated the renoprotective effect of the MSCs, while MSCs pretreated IL-10 blocking Ab showed the similar renopreotective effect. The neutralizing anti–mouse IL-10 Abs(200 µg/mouse; BioXCell) or their respective isotype control (IgG2a) was administrated intraperitoneally at 4 h before renal I/R with MSCs and serum creatinine were measured at 24h after renal I/R injury (n=4-5 per group). MSCs were also pretreated with IL-10 blocking Ab (2µg/ml for 24 h) and administered intraperitoneally 4 h before renal I/R. * p < 0.05 compared to sham, ** p < 0.05 compared to PBS + I/R + isotype IgG, **^#^** p < 0.05 compared to MSC + I/R + isotype IgG.

Because the MSC-treated CD11c^+^ cells showed increased IL-10 production, we further tested the role of IL-10 produced by the CD11c^+^ cells in mediating the renoprotective effect. While transfer of the CD11c^+^ cells from the WT mice partially restored the renoprotective effect of MSCs, transferring the IL-10-deficient CD11c^+^ cells failed to restore this beneficial effect, suggesting the important contribution of IL-10 producing CD11c^+^ cells in mediating the renoprotective effect of MSCs (Figure 9). To exclude the possibility that IL-10 production by MSCs exert renoprotective effect, we also performed experiments using IL-10 blocking antibody (Ab). We observed that administration of MSCs that were pretreated with IL-10 blocking Ab as previously described [20] showed the similar renoprotective effect. However, blocking IL-10 in MSC treated IR mice completely mitigated the renoprotective effect, suggesting that it is not IL-10 from MSC that exerts renoprotective effect (Figure 10).

## Discussion

In this study, we showed that the renoprotective effect of MSCs is partially mediated by their immune modulatory effect, possibly through direct interaction with CD11c^+^ cells in I/R-induced AKI. Splenic CD11c^+^ cells co-cultured with MSCs showed a regulatory phenotype and MSC-induced suppression of T-lymphocyte proliferation and expansion of Tregs were all inhibited when the CD11c^+^ cells were depleted. Although pretreatment with the MSCs markedly attenuated kidney injury, depletion of the CD11c^+^ cells partially mitigated the renoprotective effect of the MSCs, thereby suggesting that these cells play a critical role in mediating the beneficial effect of MSCs in I/R injury.

The transdifferentiation-independent paracrine effect of MSCs in ischemic or nephrotoxic AKI has been previously shown. However, the immune modulatory effect of MSCs has recently gained more attention, and their possible contribution to the beneficial effect of MSCs in a variety of injury models is being suggested. As part of the immune modulatory effect, their role in modulating DC phenotype has been shown recently. Jiang et al. observed that MSCs inhibited the in vitro differentiation and function of CD14^+^ monocyte-derived DCs [21]; Furthermore, the capacity of MSCs to induce a novel, Jagged-2-dependent regulatory DC population has also been reported [17]. Huang et al. observed that kidney-derived MSCs modulate DCs into immature, regulatory cells, and co-transplantation of MSC-modulated DCs significantly delayed the rejection in islet transplantation [22]. All these reports, that demonstrated the possible MSC-DC interactions, led us to hypothesize that the MSC-induced renoprotective effect in I/R injury might also be partially mediated by their interaction with DCs.

DCs in I/R injury were initially reported to be detrimental as they produce various proinflammatoy cytokines [23]. However, DCs possess phenotypic plasticity [24,25], and a subsequent study from our laboratory clearly showed the regulatory role of CD11c^+^ cells in the recovery phase that participates in the repair process in I/R-induced AKI [18]. The immune regulatory and renoprotective function of DCs have also been recently shown using crescentic glomerulonephritis animal models [26–28].

In this study, we first examined the effect of MSCs on the phenotype of CD11c^+^ cells. CD11c is a well-known marker of DCs; however, because it can also be expressed in macrophages, and simultaneous macrophage depletion can also occur in CD11c-DTR system, we designated these cells as CD11c^+^ cells, not DCs.

Splenic CD11c^+^ cells co-cultured with MSCs acquired more immature phenotype ; produce more IL-10, increased CD11b expression, decreased CD11c and CD80 expression that is comparable with other recent finding by Jhang et al [17]. These phenotypic changes observed in the MSC-treated cells are considered to be mediated through a direct cell–cell contact mechanism, because the CD11c^+^ cells that were physically separated in the trans-well system did not reproduce these results. In addition to the in-vitro effect of MSCs on the CD11c^+^ cells, we also observed phenotypic alterations of the CD11c^+^ cells in the MSC-treated I/R mice. IL-10 production from the splenic CD11C^+^ cells in the MSC-treated mice was significantly higher than that observed in the vehicle-treated mice and the percentage of kidney CD11c^+^ DCs that co-expressed the maturation marker was significantly decreased in the MSC-treated I/R kidneys. All these results show that the MSCs induced a phenotypic change of CD11c^+^ cells with regulatory function.

In addition to MSCs direct effect on phenotype of CD11c^+^ cells, we also assessed whether CD11c^+^ cells are involved in MSCs immune modulatory effect on T-cell function. First, we found that MSCs suppressed normal T cell proliferation. More importantly, the suppression by MSCs were partially abrogated by depletion of CD11c^+^ cells, suggesting the important contribution of CD11c^+^ cells in MSCs induced inhibitory effect on T cell function. CD4^+^ T cells are known to infiltrate into the ischemic kidney and play an important role in I/R injury by producing a variety of cytokines such as IFN-γ [29,30]. The effects of mononuclear phagocytes on MSCs and other stromal cell-induced suppressions of T-cell proliferation have been shown in several other studies. González-Rey et al. (2009) showed that the depletion of CD14^+^ monocytes from peripheral blood mononuclear cells significantly abrogated the suppressive effect of human adipose tissue-derived stromal cells (hASCs) on T-cell proliferation [9]. They also observed that adding monocytes dose-dependently restored the suppressive activity of hASCs on T cells as well as IFN-γ secretion in an hMSCs-CD4 co-culture system. All these results suggest the important contribution of mononuclear phagocytes/CD11c^+^ cells in mediating MSCs-induced inhibitory effects of T-cell activation. Although we used a somewhat different approach that tested the MSCs anti-proliferative effect with using CD11c^+^ cell depleted splenocytes-MSC co-culture system, we also observed that the inhibitory effect of MSCs on T-cell proliferation induced by anti-CD3 and anti-CD28 antibodies was completely abrogated when splenocytes that were depleted in CD11c^+^ cells were used.

The CD4^+^ CD25^+^ Foxp3^+^ Tregs are important in the induction of immune tolerance and suppression of the innate immune response, exerting a renoprotective function in I/R injury [31,32]. There have been several studies showing that MSCs induce Treg expansion [9–12], and therefore, we first tested whether MSCs induce expansion of Tregs, and if so, whether CD11c^+^ cells play critical in MSC-induced expansion of Tregs. In our in vitro and in vivo experiment, we observed that Tregs were expanded by MSC treatment and it was partially inhibited when CD11c^+^cells were depleted using CD11c DTR transgenic mice. These results could also suggest the important contribution of CD11c^+^ cells in MSCs induced Treg expansion.

Next, we performed an in vivo experiment using CD11c^+^-DTR transgenic mice to determine the role of CD11^+^ cells in renoprotection by MSCs. Interestingly, we first observed that depletion of CD11c^+^ cells before I/R did not affect the initial kidney injury. This is in contrast with previous study by Li et al. that showed the significant renoprotective effect of depletion of CD11c^+^ cells in IRI [33]. However, subsequent studies have shown no beneficial effect [34–36]. Although the exact reasons for this discrepancy are not clear, the magnitude of simultaneous depletion of macrophages in the CD11c-DTR system, possibly due to different timing or doses of DT might be responsible. Although depleting CD11c^+^ cells did not affect kidney dysfunction following IRI, it partially abrogated the renoprotective effect of MSCs. Furthermore, a direct causal relationship between the renoprotective effects of MSCs and CD11c^+^ cells was shown by the CD11c^+^ cell adoptive transfer experiment. The partially restored renoprotective effect of the MSCs by adoptive transfer of the CD11c^+^ cells following depletion of these cells strongly suggests the important role of CD11c^+^ cells in mediating the renoprotective effect of MSCs.

Because our in-vitro experiments showed the acquisition of regulatory phenotype in CD11c^+^ cells by MSCs is likely to be important, we hypothesized that absence of IL-10 in CD11c^+^ cells might not mediate MSCs renoprotective effect.

While the adoptive transfer of the CD11c^+^ cells from the WT animals following depletion partially restored the beneficial effect of the MSCs, transferring the CD11c^+^ cells from the IL-10 KO mice failed to restore the MSC effect. These results strongly suggest that IL-10 producing CD11c^+^ cells, possibly induced by their direct interaction with MSCs, play a critical role in mediating the renoprotective effect of MSCs.

## Conclusions

In conclusion, our study, for the first time, showed that the renoprotective effect of MSCs is partially mediated by their direct interaction with CD11c^+^ cells and expansion of Tregs, that is at least in part, dependent on IL-10.

The unique characteristics of MSCs, such as low immunogenicity and immunomodulatory effects, suggest MSCs are promising tools for therapeutics and preventive strategies. A better understanding of the mechanisms of MSCs in reducing kidney injury and promoting recovery is required for the development of clinically applicable strategies for the prevention and treatment of AKI.

## Supporting Information

Figure S1 Depletion of GFP^+^ cells by diphtheria toxin administration. The treatment of the transgenic mice with the human diphtheria toxin (DT, 4 ng/g) resulted in a significant depletion of GFP^+^ cells in both spleen and kidney.
(TIF)
