# Supplementary figures and images for: CD11c+ Cells Partially Mediate the Renoprotective Effect Induced by Bone Marrow-Derived Mesenchymal Stem Cells
Source: PLoS One. 2013 Aug 6;8(8):e72544. doi: 10.1371/journal.pone.0072544 (PMC3735517; doi:10.1371/journal.pone.0072544)

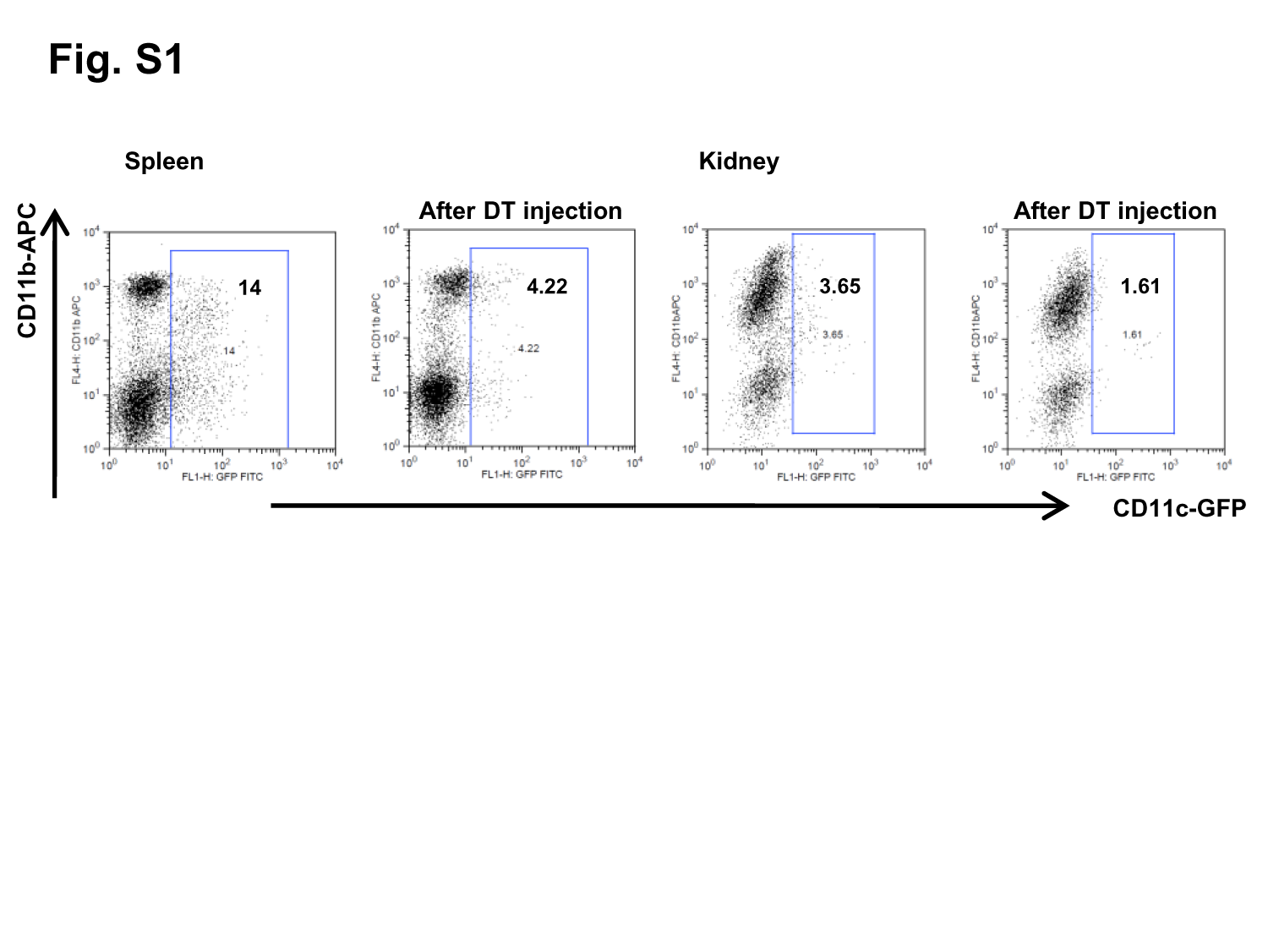

Supplement: Figure S1 — The treatment of the transgenic mice with the human diphtheria toxin (DT, 4 ng/g) resulted in a significant depletion of GFP+ cells in both spleen and kidney. (TIF) [file pone.0072544.s001.tif]
